# Influence of temperature and humidity manipulation on chicken embryonic development

**DOI:** 10.1186/s12917-014-0234-3

**Published:** 2014-10-01

**Authors:** Rute M Noiva, António C Menezes, Maria C Peleteiro

**Affiliations:** Interdisciplinary Research Centre on Animal Health, Faculty of Veterinary Medicine, University of Lisbon (CIISA/FMV/ULisboa), Lisbon, Portugal; Laboratório de Anatomia Patológica, Faculdade de Medicina Veterinária, Avenida da Universidade Técnica, Lisboa, 1300-477 Portugal

**Keywords:** Chicken, Embryonic development, Temperature, Humidity, Incubation

## Abstract

**Background:**

Temperature and relative humidity (RH) are very important factors affecting embryo development, hatchability, and posthatch performance. This study aimed at characterizing embryonic metabolic and behavioural response to a harsh incubation environment generated by manipulations (elevations and drops) in these two key factors. This study was aimed at establishing patterns of metabolic and behavioural response, as well as mortality and the development of malformations, all of which can potentially be used in monitoring incubating operations and diagnosing problems with faulty equipment.

**Results:**

Of all the parameters monitored throughout embryonic development the ones shown to be most affected were: albumen-weight to egg-weight ratio (AR); yolk-weight to egg-weight ratio (YR); embryo-weight to egg-weight ratio (ER); heart rate (HR); voluntary movements per minute (VMM); mortality rates; malformation prevalence and type.

The most significant changes in the evolution of AR and YR throughout incubation involved delay and reduction in the amplitude of the expected drop in albumen and yolk levels, reflecting lower nutrient consumption by the embryo. ER tended to grow more slowly and remain lower than the established normal, especially in embryos challenged with temperature treatments. HR and VMM were considered to be strong indicators of embryonic stress, as all treatments applied resulted in elevated heart rate and decreased embryo movement.

Mortality rates for both temperature-related treatments were higher during the first four days of incubation. Changes in relative humidity have produced less radical effects on mortality. Malformation rates were higher for embryos subjected to high incubation temperatures and were most prominently related to the abdominal wall, head, skull and limbs.

Overall, manipulations in environmental (incubator) temperature during incubation produced more drastic changes in embryo development than humidity-related manipulations, especially where mortality and malformation rates were concerned.

**Conclusions:**

This paper describes changes in embryonic metabolism and behaviour, as well as in mortality and malformation rates, in response to manipulations in environmental temperature and relative humidity. Together with further studies, these patterns may prove helpful in the diagnosis of equipment malfunctions relating to temperature or relative humidity.

## Background

Chicken embryos are poikilothermic, relying on an external source (hen or incubator) to provide the heat necessary for development and maintenance of normal metabolic functions. Therefore, temperature influences the metabolic rate of yolk and albumen mobilization and consumption and, subsequently, embryonic development throughout incubation [1,2].

Temperature may, however, have a divergent, or biphasic, effect during the course of incubation. Raising temperature initially accelerates embryonic growth and utilization of nutrients and energy from yolk and albumen, but as incubation progresses, exposure to constant high temperatures decreases embryonic growth [1].

Exposure to low temperatures early in incubation not only appears to affect embryonic heat production (by influencing embryonic metabolic rates), but is also reported to influence embryonic and post-hatch development [3]. It has been shown that, early in incubation, the metabolic rate rises in response to higher incubation temperatures whereas, prior to pipping, both chicken and duck embryos show decreased metabolic rates as the internal egg temperature exceeds 40.0°C [3].

It has been hypothesized that, if embryonic growth deviates from optimum, both post-hatch growth and organ function will be impaired [4]. Consequently, it is important to incubate eggs at a temperature that optimizes hatchability, currently defined as being between 37 and 38°C (most often between 37.5 to 37.8°C) [5-7].

As incubation (and therefore embryo development) progresses and nutrients (*e.g*., lipids) are incorporated into the embryo and metabolized, the egg loses water through evaporation. The rate of incubational water loss by the egg has an effect on embryogenesis and the total water loss influences the creation an air cell sufficiently large to allow embryonic lung ventilation after internal pipping and a successful hatch [6,8]. Embryonic mortality increases when water loss is lower than 9.1% or higher than 18.5% [6]. Dehydration can occur as a result of low relative humidity (RH) during incubation or hatching, or if a long period of time passes between hatching and removal from the hatcher (pull) [6,8]. The optimal RH range established is quite wide, between 40 and 70% RH, with the maximum hatchability obtained around 50% RH [9].

Problems with equipment maintenance, incubator cooling, airflow patterns and other conditions may cause embryos to overheat or cool down, to become overhydrated or dehydrated, negatively affecting hatchability and reducing chick quality. This study was aimed at establishing patterns of metabolic and behavioural response, as well as mortality and the development of malformations, all of which can potentially be used in monitoring incubating operations and diagnosing problems with faulty equipment.

## Methods

All eggs used in this study were first-grade specific-pathogen-free (SPF) hatching eggs (breed Lohmann Selected Leghorn-White Leghorn), obtained from a commercial supplier of Vaccine Eggs (VALO BioMedia). An OVA-EASY® Advance 380 (Brinsea) cabinet incubator with a maximum capacity of 384 eggs was used.

### Establishment of normal development patterns

Four-hundred and seventy first-grade specific-pathogen-free (SPF) hatching eggs were obtained, in two separate batches. The eggs weighed between 49.64 g and 64.1 g, (average weight 54.76 g ± 3.4 g) and were stored for less than 2 days, at 14-16°C, 75-80% relative humidity (RH), and pre-warmed at 22-24°C for 12 h before incubation.

Eggs were randomly divided and set in vertical position (blunt end up) across 6 incubator trays. Incubation conditions were as follows: from day 0 to day 18 eggs were incubated at 37.8°C, RH 50-55%; from day 18 onwards the eggs were set on the hatcher trays (in horizontal position) and incubated at 37.8°C, RH 60-65%.

A random sample of 5% of all eggs was removed from the incubator every two days of incubation (from day 0 to day 20) and submitted to the procedures described in Table 1. A PowerLux® Egg Candler (Lyon Technologies, Inc.) was used for candling. Heartrate was assessed by visual inspection upon candling from day 3 to day 5 of development and via an electronic digital egg monitor (Buddy Mk2®, Avitronics) from day 6 onwards. Voluntary movements were assessed by visual inspection upon candling. Euthanasia was performed according to the European Parliament [10,11] and the American Veterinary Medical Association (AVMA) guidelines. Briefly, embryos between days 0 and 14 of incubation were placed in a refrigerated environment (<4°C) for 4 hours prior to break-out, while embryos between days 16 and 20 were placed in a chamber with 100% environmental CO_2_ concentration for 20 minutes prior to break-out. Death was confirmed through exsanguination without maceration, in an effort to preserve embryos for future histopathological study.

**Table 1:** **Procedures applied to embryos on breakout according to incubation period**

**Procedure**	**Incubation day**		
	**0** **-** **2**	**3** **-** **15**	**15-** **20**
Weighing	x	x	x
Candling	x	x	x
Detection of embryo		x	x
Assessment of HR and VMM		x	x
Embryo euthanasia			
(<4°C, 4 h)	x	x	
(100%, CO2)			x
Detection and measurement of blastodisc/blastoderm	x		
Detection and measurement of embryo		x	x
Weighing of embryo		x	x
Weighing of the albumen, yolk and shell	x	x	x

### Trial 1 – Challenge with constant sub- or supraoptimal temperatures

Five-hundred and forty first-grade specific-pathogen-free (SPF) hatching eggs were obtained, in two equally sized batches. The eggs weighed between 50.68 g and 66.34 g (average weight 60.48 g ± 2.94 g).

Handling before incubation was performed as described for Control eggs. The first batch (Treatment A) was incubated at 38.9°C, RH 50-55% from day 0 to day 18; from day 18 onwards the eggs were set on the hatcher trays (in horizontal position) and incubated at 38.9°C, RH 60-65%. The second batch (Treatment B) was incubated at 36.7°C, RH 50-55% from day 0 to day 18; from day 18 onwards the eggs were set on the hatcher trays (in horizontal position) and incubated at 36.7°C, RH 60-65%.

A random sample of 5% of all eggs was removed from the incubator at each day of incubation (from day 0 to day 20) and treated as described for Control eggs.

### Trial 2 – Challenge with constant sub- or supraoptimal relative humidity

Five-hundred and forty first-grade specific-pathogen-free (SPF) hatching eggs were obtained. The eggs weighed between 50.66 g and 78.27 g, (average weight 62.89 g ± 5.8 g).

Handling before incubation was performed as described for Control eggs. The first batch (Treatment C) was incubated at 37.8°C, RH 60-65% from day 0 to day 18; from day 18 onwards the eggs were set on the hatcher trays (in horizontal position) and incubated at 37.8°C, RH 60-65%. The second batch (Treatment D) was incubated at 37.8°C, RH 40-45% from day 0 to day 18; from day 18 onwards the eggs were set on the hatcher trays (in horizontal position) and incubated at 37.8°C, RH 40-45%.

A random sample of 5% of all eggs was removed from the incubator every two days of incubation (from day 0 to day 20) and treated as described for Control eggs.

### Statistical analysis

The data were subjected to one-way ANOVA and to the all-pairs Tukey-Kramer-HSD test, by means of the SPSS Statistics 19 software (IBM, 2013).

### Ethical statement

This study and the project to which it pertains were designed according to the European laws that regulate laboratory animal care and use, and submitted to the Commission of Ethics and Animal Welfare (CEBEA) of the Faculty of Veterinary Medicine (University of Lisbon), which deemed this study to be beyond the sphere of application of EU Parliament Directive 2010/63/UE, relating to the protection of animals used for scientific purposes, and therefore without need of approval by the same Commission.

## Results

### Trial 1 – Challenge with constant sub- or supraoptimal temperatures

The most prominent differences in the parameters monitored for this trial were related to albumen-weight to egg-weight ratio (AR), yolk-weight to egg-weight ratio (YR), embryo-weight to egg-weight ratio (ER), heart rate (HR), voluntary movements per minute (VMM), mortality rates (MR), and malformation prevalence and type.

**Figure 1:**
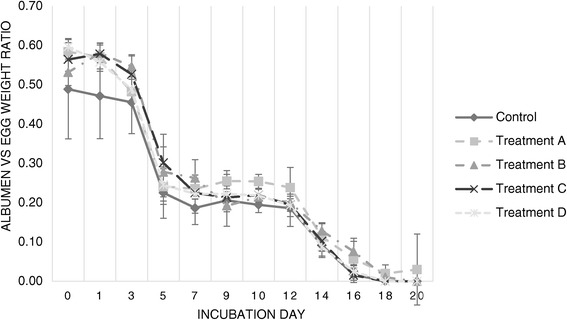
**Evolution of the albumen** **-to-** **egg-** **weight ratio throughout incubation.** Evolution of the ratio of total albumen weight in proportion to egg fresh weight throughout twenty days of incubation (values displayed are the average for each sample of viable eggs per day of sampling).

**Figure 2:**
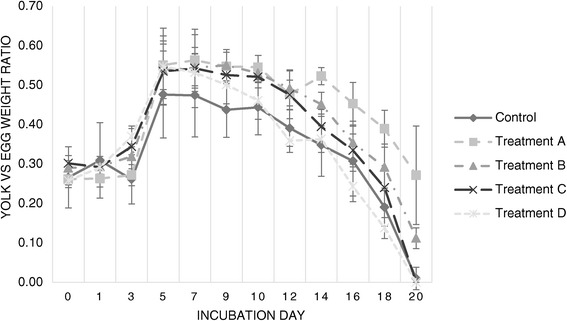
**Evolution of the yolk-** **to-** **egg-** **weight ratio throughout incubation.** Evolution of the ratio of yolk albumen weight in proportion to egg fresh weight throughout twenty days of incubation (values displayed are the average for each sample of viable eggs per day of sampling).

**Figure 3:**
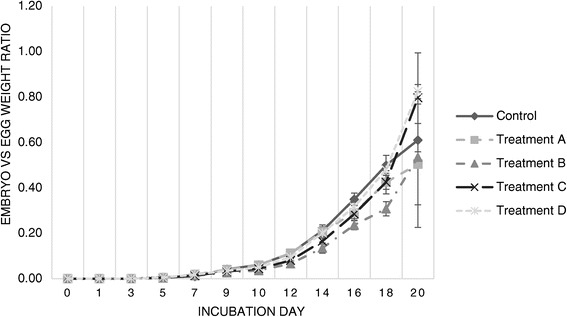
**Evolution of the embryo-** **to-** **egg-** **weight ratio throughout incubation.** Evolution of the ratio of total embryo weight in proportion to egg fresh weight throughout twenty days of incubation (values displayed are the average for each sample of viable eggs per day of sampling).

**Figure 4:**
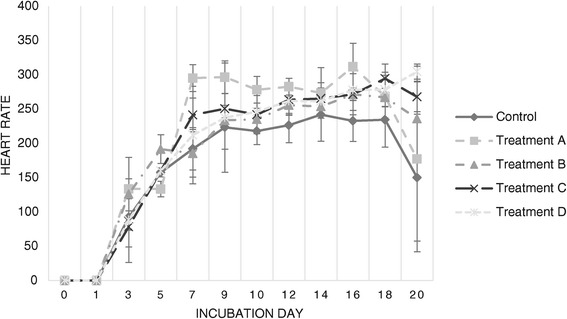
**Embryonic heart rate throughout incubation.** Average embryonic heart rate for each sample of viable embryos per day of sampling, as detected via candling and visual inspection from days 0–5, and via digital heart rate monitor from day 6 onward.

**Figure 5:**
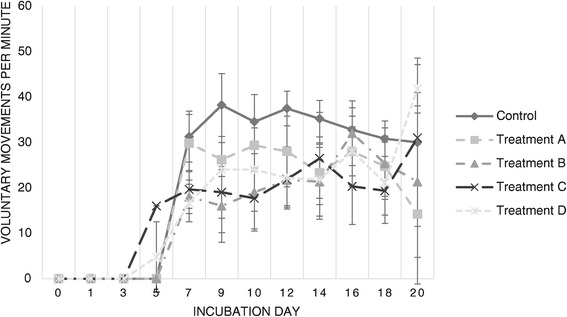
**Voluntary movements per minute throughout incubation.** Average voluntary embryonic movements for each sample of viable embryos per day of sampling, as detected via candling and visual inspection.

Although significant differences between treatments may be identified on the average values for each of the parameters mentioned (as is shown in Table 2), it may be of greater interest to observe the evolution of these values throughout incubation, illustrated in Figures 1, 2, 3, 4 and 5.

**Table 2:** **Mean values of the parameters monitored at each time**-**point**, **by treatment**

**Day**	**Treatment**	**Albumen**		**Yolk**		**Embryo**		**Heart rate**		**VMM**	
		**Mean**	**SEM**	**Mean**	**SEM**	**Mean**	**SEM**	**Mean**	**SEM**	**Mean**	**SEM**
1	Control	0.471	0.024	0.309	0.021	-	-	-	-	-	-
	A	0.571^*^	0.011	0.263	0.007	-	-	-	-	-	-
	B	0.581^*^	0.006	0.294	0.006	-	-	-	-	-	-
	C	0.578^*^	0.065	0.292	0.006	-	-	-	-	-	-
	D	0.558^*^	0.005	0.291	0.005	-	-	-	-	-	-
3	Control	0.455	0.019	0.261	0.015	-	-	92.44	10.282	-	-
	A	0.482	0.010	0.345	0.009	-	-	133.60	4.588	-	-
	B	0.455^*^	0.007	0.319^*^	0.008	-	-	126.25	13.256	-	-
	C	0.527^*^	0.014	0.272^*^	0.013	-	-	78.33	15.067	-	-
	D	0.489	0.009	0.370^*^	0.008	-	-	88.33	3.860	-	-
5	Control	0.225	0.015	0.476	0.025	0.005	0.002	157.4	1.459	-	-
	A	0.241	0.015	0.551	0.021	0.005	0.002	133.33^*^	3.771	-	-
	B	0.280	0.023	0.538	0.031	0.003	0.000	191.5^*^	7.385	-	-
	C	0.301^*^	0.022	0.535	0.021	0.004	0.000	160	0.000	16.000^*^	0.000
	D	0.245	0.013	0.547	0.031	0.005	0.000	160	0.000	4.800^*^	20.444
7	Control	0.187	0.009	0.474	0.023	0.009	0.002	191.91	6.712	31.238	1.223
	A	0.235	0.008	0.563^*^	0.020	0.019	0.000	295.00^*^	5.881	29.818	1.897
	B	0.264^*^	0.016	0.544	0.018	0.013	0.001	185.38	12.230	18.000^*^	1.309
	C	0.225	0.005	0.543	0.030	0.014	0.000	241.36^*^	7.415	19.636^*^	1.260
	D	0.222	0.014	0.532	0.014	0.018^*^	0.001	211.92	20.490	16.667^*^	1.189
9	Control	0.206	0.015	0.438	0.016	0.041	0.001	223.35	7.158	38.200	1.549
	A	0.255^*^	0.008	0.547^*^	0.011	0.039	0.001	296.46^*^	7.152	26.182^*^	1.127
	B	0.190	0.007	0.552^*^	0.017	0.026^*^	0.001	233.60	1.631	16.000^*^	3.578
	C	0.213	0.006	0.526^*^	0.006	0.031^*^	0.001	250.42	6.360	19.000^*^	1.642
	D	0.222	0.006	0.501^*^	0.015	0.035^*^	0.002	237.36	24.040	24.000^*^	4.178
10	Control	0.195	0.005	0.444	0.016	0.060	0.002	217.84	4.558	34.526	1.375
	A	0.254^*^	0.006	0.546^*^	0.010	0.060	0.002	278.11^*^	6.458	29.333	1.886
	B	0.214	0.007	0.531^*^	0.009	0.036^*^	0.001	234.88	5.642	19.000^*^	3.000
	C	0.219	0.005	0.521^*^	0.008	0.044^*^	0.001	241.83	8.038	17.667^*^	1.936
	D	0.218	0.007	0.461	0.014	0.049^*^	0.001	245.83^*^	7.872	24.000^*^	2.652
12	Control	0.186	0.010	0.391	0.001	0.111	0.003	226.41	5.473	37.455	0.813
	A	0.239^*^	0.016	0.477^*^	0.020	0.112	0.003	282.50^*^	3.804	28.000^*^	2.459
	B	0.200	0.017	0.492^*^	0.017	0.066^*^	0.005	255.57^*^	4.493	21.714^*^	2.286
	C	0.1940	0.004	0.476^*^	0.010	0.080^*^	0.002	263.54^*^	6.285	21.846^*^	1.609
	D	0.1931	0.008	0.359	0.008	0.095^*^	0.002	261.62^*^	5.579	22.154^*^	1.901
14	Control	0.092	0.007	0.348	0.018	0.211	0.006	241.70	8.6749	35.200	0.8991
	A	0.113	0.011	0.523^*^	0.007	0.207	0.006	273.46	11.143	23.273^*^	1.690
	B	0.128	0.007	0.451^*^	0.013	0.135^*^	0.009	253.67	4.794	21.333^*^	3.373
	C	0.102	0.007	0.395	0.012	0.165^*^	0.004	265.23	6.386	26.462^*^	2.812
	D	0.087	0.007	0.363	0.010	0.201	0.005	261.36	5.278	21.818^*^	2.434
16	Control	0.017	0.003	0.308	0.019	0.349	0.006	232.64	6.389	32.727	1.041
	A	0.057^*^	0.014	0.453^*^	0.017	0.289^*^	0.011	311.80^*^	10.690	28.000	2.459
	B	0.077^*^	0.014	0.353	0.022	0.236^*^	0.008	271.33	3.7298	32.000	2.921
	C	0.015	0.005	0.335	0.012	0.284^*^	0.006	271.46^*^	8.3233	20.308^*^	2.330
	D	0.025	0.004	0.243^*^	0.011	0.316^*^	0.009	277.75^*^	8.6200	28.000	1.557
18	Control	0.001	0.001	0.191	0.011	0.499	0.010	234.40	8.957	30.800	0.875
	A	0.020	0.007	0.390^*^	0.014	0.424^*^	0.015	268.09	9.353	24.727	2.005
	B	0.008	0.004	0.290^*^	0.024	0.307^*^	0.013	266.83	5.400	25.333	3.211
	C	0.000	0.000	0.240^*^	0.013	0.424^*^	0.009	294.42^*^	6.062	19.333^*^	1.544
	D	0.000	0.000	0.138^*^	0.008	0.479	0.005	278.36^*^	7.647	21.090^*^	2.702
20	Control	0.000	0.000	0.010	0.010	0.610	0.136	150.00	32.733	30.000	6.547
	A	0.030	0.030	0.042^*^	0.042	0.555	0.036	45.069	45.069	14.222	5.126
	B	0.000^*^	0.015	0.113	0.015	0.534	0.014	236.00	31.342	21.333	9.615
	C	0.000	0.000	0.000	0.000	0.796	0.010	267.75^*^	7.643	31.000	3.525
	D	0.000	0.000	0.000	0.000	0.823	0.010	304.44^*^	3.667	41.778	1.778

^*^Significant difference found (p < 0.05). All significant values are displayed in comparison to the mean of the Control group.

In Figure 1 it is possible to observe that the eggs in treatment A consistently exhibited higher AR than Controls. Treatment B eggs had limited effects on AR values.

Regarding yolk to egg weight ratio (Figure 2), eggs in both Treatment A and B consistently exhibited higher AR than Controls.

Embryo to egg ratios for Treatment A eggs showed very similar patterns and values to Control from day 0 to day 16, at which point Treatment A eggs begun to exhibit slower growth rates and ER values than the eggs in the Control group. Treatment B eggs exhibited similar results, with lower ER values that start on day 9 of incubation.

Heart rate (Figure 4) was consistently higher than control values for both challenged groups, particularly from day 5 onward. In contrast, VMM were lower than those observed for the Control group (Figure 5), prominently from day 7 onward.

**Figure 6:**
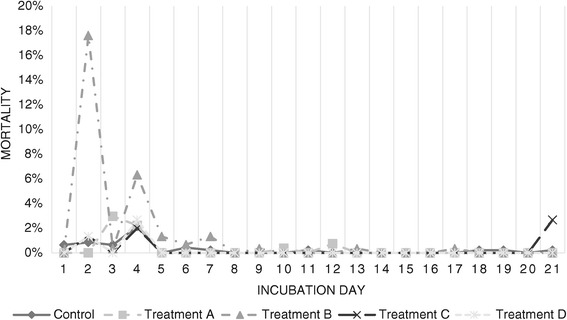
**Mortality distribution for each treatment according to incubation period.** Mortality curves for each treatment throughout 20 days of incubation. Values are displayed as percentage of embryos dead at each day of incubation (time of death established by visual inspection of the embryo and comparison with normal embryos) per total of fertile eggs incubated.

Mortality rates (Figure 6) were higher during the first 4 days of incubation for embryos in Treatment A, with a second mortality peak between days 13 and 17. Mortality rates in Treatment B were higher during the first 12 days of incubation.

**Figure 7:**
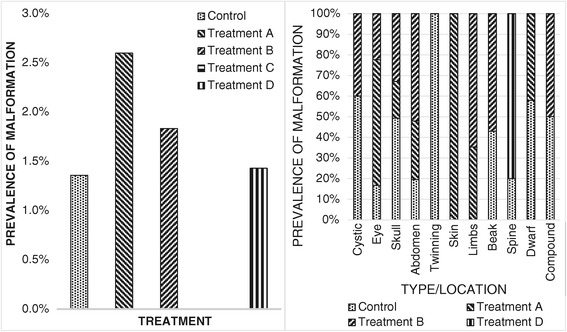
**Malformation rates by treatment and by organ/** **tissue affected.** On the left: Percentage of malformation occurrence by treatment. Values are displayed as percentage of embryos carrying one or more malformations per total of fertile eggs incubated. On the right: Percentual distribution of systems, organs and tissues prefferentially affected by malformations, in relation to each of the treatments applied.

Malformation rates (Figure 7) were highest for embryos subjected to Treatment A but also significantly higher for embryos in Treatment B than in the Control group. Malformations were most prominently related to the abdominal wall (celosomia) for both treatments. Head and skull malformations (anencephaly and exencephaly) and limb malformations (plantigrady and polymelia) were also identified for both treatments. Other malformations observed were related to stunted body growth (dwarfism) and localized subcutaneous edema for embryos under Treatment A; and eye (unilateral eye duplication, microphthalmia and anophthalmia) and beak malformations (micrognathia) for embryos in Treatment B. Compound malformations (multiple alterations in a single embryo) were a common event whenever malformations were present.

### Trial 2 – Challenge with constant sub- or supraoptimal relative humidity

The most prominent differences in the parameters monitored for this trial were related to YR, HR, VMM, mortality rates, and malformation prevalence and type.

Yolk ratio values for Treatment C remained above Control YR values throughout incubation. Treatment D eggs exhibited YR values that were consistently higher than Control YR values, except for the last days of incubation (days 18–20), when YR values were significantly lower than those registered for Control eggs.

Both humidity-related treatments had limited effects on ER. Heart rate was consistently higher than control values for both challenged groups, particularly from day 10 onward.

Both Treatment C and Treatment D embryos exhibited early onset of voluntary movement (on day 5 instead of the usual day 6–7), albeit consistently presenting VMM values lower than the embryos in the Control group.

Mortality rates were higher during the first 4 days and on the last day of incubation for embryos in Treatment C. Embryos incubated under Treatment D exhibited greater mortality rates during the first 4 days of incubation. No malformations were observed in embryos challenged with Treatment C. Malformations present in embryos subjected to Treatment D included head and skull malformations (exencephaly), abdominal wall malformations (celosomia), and posterior twinning (katadidymus).

## Discussion

Environmental conditions during incubation have a prominent effect on embryonic development.

After oviposition, embryonic development in birds is dependent upon the external temperature to which the egg is exposed and will vary in response to changes in that temperature, even if the rate of growth appears to be narrowly regulated within a species [5,7]. Developmental processes should proceed most efficiently under optimum environmental conditions as embryos are sensitive to lower or higher incubation temperatures, with lower incubation temperatures generally retarding and higher incubation temperatures accelerating growth, development and nutrient usage [12]. The risk of dehydration or overhydration is another hazard to be taken into consideration. This can be influenced by relative humidity during incubation and hatching, and incubation at a lower RH has been reported by some authors to reduce chick body weight [6,9,13].

All of the parameters monitored were affected by the manipulations made in this study, suggesting specific ways in which the embryo responds to changes in the incubation environment.

Albumen and yolk are the two main sources of nutrients for the avian embryo, and without access to any other sources than those provided by the egg itself for its nourishment [14,15], AR and YR were considered, for the purposes of this study, as monitors of nutrient consumption during embryonic development. Thus, the negative effects on AR and YR were interpreted as lowered embryonic metabolism in response to the stress caused by less-than-ideal incubation conditions imposed on the eggs.

Embryo-to-egg weight ratio, on the other hand, was considered a monitor for embryonic growth, which tended to evolve more slowly and remain lower than the established normal, especially in embryos challenged with temperature treatments, namely Treatment B.

The two remaining altered parameters – heart rate and voluntary movements per minute – were considered to be strong indicators of embryonic stress, as all treatments applied resulted in elevated heart rate and decreased embryo movement.

Taking our results and other published reports into consideration [12,16,17], one can hypothesize that the results obtained reflect a chain of reactions from the embryo to a stressful incubation environment. As thermal or hydric stress is registered, the embryo responds by lowering its metabolism (and henceforth its consumption of both yolk and albumen) in an attempt to compensate for the limiting and harsh environmental conditions. Similar observations have been communicated by Uni *et al*. (2014) [18].

Although exposure to constant high temperatures is known to increase embryonic metabolism, this effect seems to be biphasic, with metabolic acceleration being restricted to the first half of incubation (until day 9), during which heat production by the embryo is minimal.

The final result that would be expected is that of generally lighter embryos and a batch of hatchlings with poor uniformity in bodyweight, especially when temperature constitutes the limiting factor. However, unlike in other published works [12,17], we did not perceive, in this study, a significant effect on embryo-to-egg weight ratios for embryos incubated under constant high temperatures.

Mortality rates for both temperature-related treatments were higher during the first four days of incubation, when the organs of the main systems and apparatuses are developing. Temperature changes produced much more prominent changes on embryo mortality, particularly when temperature dropped 1.1 degrees Celsius below the established ideal temperature of 37.8°C. These results are in agreement with previously published works [4,6,19].

Changes in relative humidity produced less radical effects on mortality, with values remaining within those defined as normal. The only exception was for a sudden increase in mortality at the end of incubation for embryos challenged with Treatment C, most likely caused by overhydration.

Malformation rates were higher for embryos subjected to high incubation temperatures and were most prominently related to the abdominal wall, head, skull and limbs for both temperature-related treatments. Compound malformations were a common event whenever malformations were present, suggesting the possibility of multiple lesions to the stem-cell population caused by thermal stress, which then evolved into different sequelae in various organs and systems or, alternatively, affected rapidly growing key support structures (*e.g*., blood vessels) due to the accelerated growth of the embryo during the first few days of incubation. Malformations present in embryos subjected to relative humidity-related treatments were less frequent or not present (for high constant relative humidity embryos).

## Conclusions

This paper describes changes in embryonic metabolism and behaviour, as well as in mortality and malformation rates, in response to manipulations in environmental temperature and relative humidity. Overall, manipulations in environmental (incubator) temperature during incubation produced more drastic changes in embryo development than humidity-related manipulations, especially whenever mortality and malformation rates were concerned.

Although this study was limited to a relatively small sample of animals (especially when the number of eggs hatched per incubator and hatchery in a normal, industrial environment are considered), the results obtained can shed some light on patterns of embryonic response to changes in incubation environment. Even if there is no one pathognomonic change in response to each of the treatments concerned in this study, applying these observations to a larger sample of eggs could refine these patterns further and prove to be an important addition to the diagnostic tools available to farmers.

## References

[1] Romanoff AL, Romanoff AJ (1972). Pathogenesis of the Avian Embryo; An Analysis of Causes of Malformations and Prenatal Death [by] Alexis L. Romanoff, with the Collaboration of Anastasia J. Romanoff.

[2] Deeming DC, Ferguson MWJ (1991). Egg Incubation: Its Effects on Embryonic Development in Birds and Reptiles.

[3] Janke O, Tzschentke B, Hochel J, Nichelmann M (2002). Metabolic responses of chicken and muscovy duck embryos to high incubation temperatures. Comp Biochem Physiol A Mol Integr Physiol.

[4] Lourens A, van den Brand H, Meijerhof R, Kemp B (2005). Effect of eggshell temperature during incubation on embryo development, hatchability, and posthatch development. Poult Sci.

[5] Fasenko GM, Wilson JL, Robinson FE, Hardin RT (1999). Effects of Length of Egg Nest Holding Time and High Environmental Temperatures on Prestorage Embryonic Development, Survival, and Hatchability of Broiler Breeders. J Appl Poultry Res.

[6] van der Pol CW, Van Roovert-Reijrink IAM, Maatjens CM, van den Brand H, Molenaar R (2013). Effect of relative humidity during incubation at a set eggshell temperature and brooding temperature posthatch on embryonic mortality and chick quality. Poult Sci.

[7] Yalcin S, Siegel PB (2003). Exposure to cold or heat during incubation on developmental stability of broiler embryos. Poult Sci.

[8] Burnham MR, Peebles ED, Gardner CW, Brake J, Bruzual JJ, Gerard PD (2001). Effects of incubator humidity and hen age on yolk composition in broiler hatching eggs from young breeders. Poult Sci.

[9] Bruzual JJ, Peak SD, Brake J, Peebles ED (2000). Effects of relative humidity during incubation on hatchability and body weight of broiler chicks from young breeder flocks. Poult Sci.

[10] COMMUNITIES COTE (1986). Council Directive 86/609/EEC of 24 November 1986 on the approximation of laws, regulations and administrative provisions of the Member States regarding the protection of animals used for experimental and other scientific purposes. Book Council Directive 86/609/EEC of 24 November 1986 on the approximation of laws, regulations and administrative provisions of the Member States regarding the protection of animals used for experimental and other scientific purposes. City.

[11] PARLIAMENT E (2010). DIRECTIVE 2010/63/EU of the European Parliament and of the Council of 22 September 2010 on the protection of animals used for scientific purposes. Book DIRECTIVE 2010/63/EU of the European Parliament and of the Council of 22 September 2010 on the protection of animals used for scientific purposes. City.

[12] Hulet R, Gladys G, Hill D, Meijerhof R, El-Shiekh T (2007). Influence of egg shell embryonic incubation temperature and broiler breeder flock age on posthatch growth performance and carcass characteristics. Poult Sci.

[13] Bruzual JJ, Peak SD, Brake J, Peebles ED (2000). Effects of relative humidity during the last five days of incubation and brooding temperature on performance of broiler chicks from young broiler breeders. Poult Sci.

[14] Bellairs R, Osmond M (2005). The Atlas of Chick Development.

[15] Patten BM (1971). Early Embryology of the Chick.

[16] Molenaar R, Hulet R, Meijerhof R, Maatjens CM, Kemp B, van den Brand H (2011). High eggshell temperatures during incubation decrease growth performance and increase the incidence of ascites in broiler chickens. Poult Sci.

[17] Leksrisompong N, Romero-Sanchez H, Plumstead PW, Brannan KE, Brake J (2007). Broiler incubation. 1. Effect of elevated temperature during late incubation on body weight and organs of chicks. Poult Sci.

[18] Uni Z, Yadgary L, Yair R, Svihus B (2014). The nutritional limitations and requirement of the breeder progeny. 14th European Poultry Conference; June, 24th; Stavanger.

[19] Lourens A (2008). Embryo Temperature during Incubation: Practice and Theory.

